# Evaluation of Radius Fracture Repair With Critical-Sized Bone Defects Using Polypropylene Surgical Mesh in Rats

**DOI:** 10.1155/aort/7262524

**Published:** 2025-06-04

**Authors:** Asrin Emami, Seyed Hadi Kalantar, Asma Mafhumi, Hiva Saffar, Iman Menbari Oskouie

**Affiliations:** ^1^Iranian Tissue Bank and Research Center, Tehran University of Medical Sciences, Tehran, Iran; ^2^Joint Reconstruction Research Center, Tehran University of Medical Sciences, Tehran, Iran; ^3^Center for Orthopedic Trans-Disciplinary Applied Research, Tehran University of Medical Sciences, Tehran, Iran; ^4^Department of Clinical Pathology, Central Laboratory, Imam Hospital Complex, Tehran University of Medical Sciences, Tehran, Iran; ^5^Urology Research Center, Tehran University of Medical Sciences, Tehran, Iran

## Abstract

Bone fractures involving critical-sized defects pose a significant challenge in orthopedic surgery, often requiring innovative strategies to promote bone regeneration. This study aimed to evaluate the effectiveness of polypropylene surgical mesh in repairing critical-sized radius bone defects in a rat model. Treatments included autologous grafts and a combination of mesh and graft, compared with an untreated control group. After 6 weeks, X-ray and CT scan analyses revealed significant bone healing and callus formation in the treated groups, with the graft + mesh group showing the most pronounced improvement. Histomorphometric analyses demonstrated that the mesh scaffold significantly enhanced new bone formation, osteoblast and osteocyte counts, and bone microarchitecture compared with grafts alone. These findings suggest that mesh scaffolds offer superior osteogenic potential and could provide a promising adjunct for treating critical-sized bone defects. Future studies should explore optimized mesh designs and the interplay between osteogenesis and angiogenesis to improve clinical outcomes.

## 1. Introduction

Bone fractures, particularly those involving critical-sized defects, pose a significant challenge in the field of orthopedic surgery [1]. These defects, typically defined as gaps that do not heal without intervention, require innovative strategies to promote bone regeneration and restore functional integrity [2]. Among various long bones, the radius is frequently studied due to its propensity for injuries, particularly in cases of trauma or disease that lead to significant bone loss [3]. The complexity of treating radius fractures with critical-sized defects is compounded by the need for materials and methods that not only stabilize the fracture but also facilitate the repair and regeneration of the bone tissue [4].

Traditional approaches to treating critical-sized bone defects include autografts, allografts, and synthetic bone substitutes [5]. Autografts, while considered the gold standard due to their osteogenic, osteoinductive, and osteoconductive properties, are limited by donor-site morbidity, limited availability, and the potential for additional surgical complications [6]. Allografts, although more readily available, carry the risk of immune rejection [7] and disease transmission [8]. Synthetic substitutes, on the other hand, offer a more controlled and customizable option but often lack the biological activity necessary to fully support bone regeneration. Therefore, there is a compelling need for alternative methods that combine mechanical support with enhanced biological healing properties [9].

In recent years, the use of surgical meshes as adjuncts in bone repair has gained attention. These materials, traditionally employed in soft tissue reinforcement, hernia repairs [10], and other surgical applications, offer a unique combination of mechanical strength, biocompatibility [11], and the potential for modification to enhance bone regeneration [12, 13]. Polypropylene surgical mesh, a widely used material in various surgical procedures, has emerged as a promising candidate for bone defect repair due to its strength, flexibility, and relatively inert biological profile [14]. When used in conjunction with bone grafts or other osteoconductive materials, polypropylene mesh may serve as a scaffold that supports bone regeneration while providing the necessary mechanical stability to the fracture site [15].

Despite the potential benefits, the application of polypropylene surgical mesh in orthopedic repair, particularly in the context of critical-sized bone defects, remains underexplored. Previous studies have primarily focused on its use in soft tissue applications [16], with limited research addressing its role in bone healing. Moreover, the specific impact of polypropylene mesh on bone regeneration, especially in the challenging environment of critical-sized defects in long bones like the radius, has not been comprehensively evaluated [17].

The present study aims to fill this gap by evaluating the effectiveness of polypropylene surgical mesh in the repair of radius fractures with critical-sized bone defects in a rat model. Rats provide a well-established model for studying bone healing and fracture repair, offering insights that are often translatable to human clinical scenarios. By assessing parameters such as bone regeneration, biomechanical strength, and histological characteristics, this research seeks to determine whether polypropylene mesh can enhance the repair process in a critical-sized defect scenario.

This investigation is particularly significant given the growing need for improved treatment strategies in orthopedic surgery. Should polypropylene surgical mesh prove effective in this context, it could represent a novel, accessible, and cost-effective adjunct to current bone repair methods. The results of this study may not only contribute to the understanding of bone healing mechanisms but also pave the way for the development of new clinical applications for surgical meshes in orthopedics.

## 2. Materials and Methods

Ten male adult rats weighing 250–300 g, maintained under adequate conditions at the Iranian Tissue Bank and Research Center, were used. This study adheres to the Animal Research: Reporting In Vivo Experiments (ARRIVE) guidelines to ensure transparency and reproducibility in the reporting of animal research. All procedures involving animal models were conducted in compliance with the ethical guidelines set by the Ethics Committee at the Iranian Tissue Bank and Research Center (IR.TUMS.AEC.1402.049). This study complies with the ethical principles outlined in the Declaration of Helsinki although it involves animal models. Every effort was made to minimize animal suffering and reduce the number of animals used.

### 2.1. Experimental Design and Surgery

Ten rats were used in this study, divided into three groups: control, graft, and graft + mesh. Each rat had its left side as the control and the right side assigned to one of the following experimental groups: graft or graft + mesh. Each graft or graft + mesh group was repeated five times. The radius defect model was created in rats to study bone regeneration and healing. Under general anesthesia, achieved with ketamine–xylazine (Teb Gostaresh, Tehran, Iran), both forelimbs were prepared for surgery. A sterile technique was maintained throughout the procedure. A longitudinal incision was made along the cranial aspect of the forelimb to expose the radius bone. Using a bone saw (Meyer-Haake, Berlin, Germany), a critical-sized defect, typically 5 mm in diameter, was precisely created in the cranial aspect of the radius. This defect size was selected to be large enough to prevent spontaneous healing and allow for the assessment of the experimental treatments [18]. The surgical site was thoroughly irrigated with sterile saline to remove debris, and the defect area was carefully examined to ensure that it was properly prepared.

The iliac bone harvesting procedure was performed under sterile conditions after anesthetizing the rats. A small incision was made over the iliac crest, and the underlying muscles were carefully dissected and retracted to expose the bone. The periosteum was then elevated, and a portion of the iliac crest was excised using a bone rongeur (Teb Gostaresh, Tehran, Iran). Following the creation of the defect and harvesting of the iliac crest, the experimental treatments were applied according to the group assignments. In the graft group, an autologous iliac crest graft was inserted into the defect. In the graft + mesh group, both the iliac crest graft and polypropylene mesh (Tehran Teb, Tehran, Iran) were used in combination. The control group was left with the untreated defect. The incision was then closed in layers using 4–0 silk (Tehran Teb, Tehran, Iran) sutures. Postoperatively, rats received analgesics to manage pain and were monitored for signs of infection or complications. This model allowed for a comprehensive evaluation of bone healing and the efficacy of different treatments in a controlled experimental setting. Postoperative analgesia was administered using buprenorphine (0.05 mg/kg, subcutaneously, every 12 h for 48 h) to manage pain following surgery.

### 2.2. Plain Radiography

Plain radiographs of the radius were captured in the anteroposterior direction (26 kVp, 12 s; Faxitron, Japan). The images were then quantitatively analyzed using NIH Image Analysis software. In this research, bone union was assessed using the RUST score. This scoring system evaluates the presence or absence of callus and the visibility of the fracture line across four cortices visible on both anteroposterior and lateral radiographs. Kooistra et al. confirmed the reliability and validity of the Radiographic Union Scale in Tracking (RUST) for assessing human long bone unions [19].

### 2.3. Computed Tomography (CT) Scan

Prior to each bone scan, a calibration scan was conducted using a three-point calibration phantom that spanned the density range from air to cortical bone. An adaptive thresholding technique was employed to distinguish between bone and nonbone. For CT measurements, three-dimensional reconstructions were generated using the built-in software. The volume of interest (VOI) was a fixed cylinder with a diameter of 9.6 mm and a height of 4 mm, encompassing the entire scanned volume of the fracture callus. A constrained three-dimensional (3D) Gaussian filter was applied to partially reduce noise in the volumes.

The following histomorphometric analyses were performed using a direct 3D approach: (i) total volume of callus (TV, mm^3^); (ii) new bone volume (BV, mm^3^); (iii) total connectivity (Conn), which measures the number of connections in a structure and indicates the maximum number of branches that can be broken before the structure splits into two parts; (iv) trabecular number (Tb.N); (v) trabecular thickness (Tb.Th); and (vi) trabecular spacing (Tb.Sp). These parameters were used either as direct overall measures or expressed as a percentage of the tissue volume.

### 2.4. Histological Analyses

The radii were fixed in 10% buffered formalin (Sigma-Aldrich, St. Louis, MO, USA) and then decalcified using a solution containing 4% hydrochloric acid (Merck KGaA, Darmstadt, Germany) and 4% formic acid (Sigma-Aldrich, St. Louis, MO, USA) for 36 h. After decalcification, the samples were embedded in paraffin and sectioned at a thickness of 5 μm. The sections were stained with hematoxylin and eosin (H&E) (Tehran Teb, Tehran, Iran). For analysis, three sections were selected from the initial, middle, and terminal regions of each sample. Systematic random fields were photographed, starting from one edge of the grafted scaffold and extending to the opposite edge. Quantitative analysis of the tissue was performed using ImageJ software, which assessed the numbers of osteoblasts, osteocytes, and osteoclasts, as well as the areas occupied by bone, connective tissue, adipose tissue, and blood vessels.

### 2.5. Postoperative Condition

All animals remained in good health throughout the study period. The mean final body weight was 275 ± 15 g. Normal ambulation and feeding behavior were observed in all rats. No signs of infection, wound dehiscence, or abnormal swelling were noted although two animals experienced mild, transient swelling at the surgical site, which resolved within 3 days without intervention.

### 2.6. Statistical Analysis

Statistical analyses were performed using GraphPad Prism (Version 9.0). The Shapiro–Wilk test was applied to assess the normality of data distribution. For data that followed a normal distribution, one-way analysis of variance (ANOVA) was conducted, followed by Tukey's post hoc test to determine differences between groups. For data that did not follow a normal distribution, the Kruskal–Wallis test was performed, followed by Dunn's multiple comparisons test. Statistical significance was defined as *p* value < 0.05.

## 3. Results

**Figure 1:**
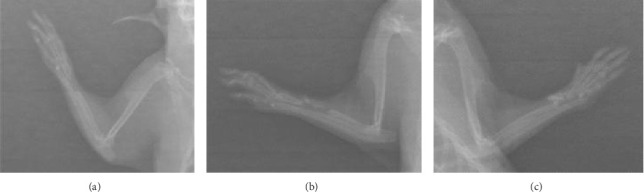
X-ray radiographs of fractured radius at 6 weeks postfracture in various groups. (a) Control, (b) graft, and (c) graft + mesh.

After 6 weeks, X-ray analyses revealed that the fracture line persisted in the control group whereas it was almost indiscernible in the treated groups (Figure 1). There was no callus formation in the control group, but significant callus formation occurred in the graft and graft + mesh groups.

The RUST scores assess cortical bone continuity and callus formation. Specifically, the control group achieved an average score of 5.1 ± 0.78 points, the graft group obtained 9.10 ± 3.74 points, and the graft + mesh group reached 11.5 ± 2.17 points. These results suggest that the graft + mesh group experienced significantly greater progress in bone union compared with the graft group (*p* < 0.05).

**Figure 2:**
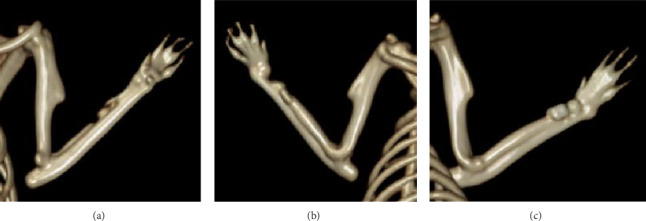
Computed tomography (CT) scan images of fractured radius at 6 weeks postfracture in various groups. (a) Control, (b) graft, and (c) graft + mesh.

In terms of qualitative analysis, 3D reconstructions of CT scans indicated a greater presence of bony callus in the treated groups compared with the control group at the 6-week mark. Specifically, the mesh combined with the graft group exhibited more substantial bony callus formation than the group receiving only the graft. CT imaging also exhibited the remodeling of the calcified callus, showing the fracture gap remaining in the control group at 6 weeks postfracture, but it was nearly nonexistent in the treated groups, particularly the graft + mesh group (Figure 2).

On a quantitative level, at 6 weeks postfracture, the TV was 4.1% greater in the graft + mesh group compared with the mesh-only group (*p* < 0.01). The BV, along with metrics such as the ratio of BV to TV (BV/TV), Conn, Tb.N, and Tb.Th, followed a similar trend to the TV at this time point (Table 1).

These quantitative findings demonstrate that combining mesh with graft notably enhanced the histomorphometric parameters in the rat radius, surpassing the effects seen with graft alone.

**Figure 3:**
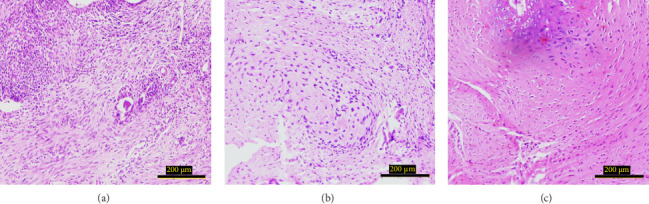
Histological sections of bone. (a) Control, (b) graft, and (c) graft + mesh. Scale bars: 200 μm.

The study investigated the effects of different treatment methods—control, graft, and graft + mesh—on capillary, adipose, connective tissue, dead bone, and new bone area formation, as well as cellular counts (osteoblasts, osteocytes, and osteoclasts). The results demonstrated significant differences in both histological (Figure 3) and cellular parameters across the three groups.

**Figure 4:**
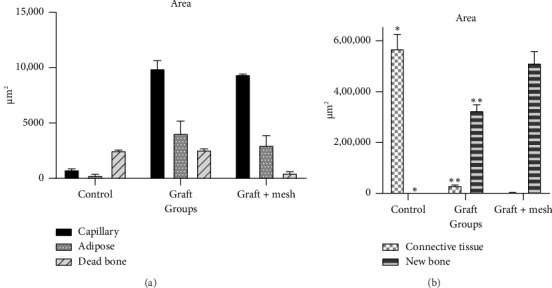
Comparison of capillary, adipose, and dead bone (a) and connective tissue and new bone formation area (b) in control, graft, and graft + mesh groups. ^∗^Significant difference with graft and graft + mesh at the same area (*p* < 0.0001). ^∗∗^Significant difference with graft + mesh at the same area (*p* < 0.0001).

Both the graft and graft + mesh groups demonstrated a statistically significant increase in new bone formation and a decrease in connective tissue compared with the control group (*p* < 0.0001). When comparing the graft and mesh groups, significant differences were observed in new bone formation and connective tissue. The graft + mesh group exhibited superior bone formation compared with the graft group (*p* < 0.0001), suggesting that the mesh material was more conducive to osteogenic processes than the graft scaffold alone. No significant difference was seen in capillary, adipose, and dead bone areas between groups (Figure 4).

**Figure 5:**
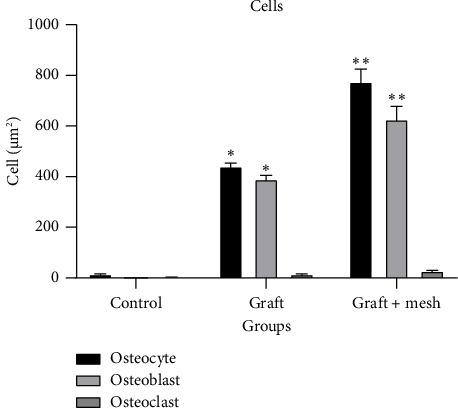
Comparison of osteocytes, osteoblasts, and osteoclasts in control, graft, and graft + mesh groups. ^∗^Significant difference with control and mesh. ^∗∗^Significant difference with graft.

Both the graft and graft + mesh groups exhibited a significantly higher count of osteoblasts and osteocytes compared with the control group (*p* < 0.0001). A direct comparison between the graft and graft + mesh groups revealed significant differences in the osteoblast and osteocyte counts (*p* < 0.0001). The graft + mesh group showed a higher number of osteoblasts and osteocytes. In addition, there was no significant difference in the number of osteoclasts between the groups (Figure 5).

## 4. Discussion

The findings from this study underscore the significant impact of combining a mesh with a graft on bone healing and callus formation in a rat fracture model. After 6 weeks, X-ray analyses revealed that the fracture line persisted in the control group, indicating delayed or incomplete healing whereas it was nearly indiscernible in the treated groups. This observation suggests that both the graft and graft + mesh treatments facilitated more effective bone repair, with the latter showing a particularly pronounced effect.

The absence of callus formation in the control group further highlights the inadequacy of natural healing in this model without intervention. In contrast, the significant callus formation observed in both the graft and graft + mesh groups emphasizes the role of these treatments in promoting osteogenesis. Notably, the graft + mesh group achieved the highest RUST scores, indicating superior cortical bone continuity and callus formation compared with the graft-alone group. The statistically significant difference (*p* < 0.05) between the two treated groups suggests that the mesh provides additional mechanical stability [20] and possibly enhances the biological environment [21] for bone regeneration [16].

The 3D CT reconstructions provided further qualitative evidence of the benefits of the combined treatment. The greater presence of bony callus in the treated groups, particularly in the graft + mesh group, aligns with the X-ray findings and supports the hypothesis that the mesh enhances the effectiveness of the graft. The nearly complete remodeling of the calcified callus in the treated groups, compared with the persistent fracture gap in the control group, illustrates the accelerated and more complete healing process facilitated by these interventions [22].

Quantitatively, the analysis at 6 weeks postfracture revealed that the TV was 4.1% greater in the graft + mesh group compared with the graft-only group. This increase in TV, along with the enhanced BV and other histomorphometric parameters such as the BV/TV ratio, Conn, Tb.N, and Tb.Th, indicates that the addition of the mesh not only increases the overall volume of new bone but also improves the quality of the bone formed. These improvements in bone microarchitecture are critical for long-term bone stability and function [23].

The superior outcomes observed in the graft + mesh group can be attributed to several factors. The mesh likely provides a scaffold that supports and guides new bone growth, offering a physical structure that helps maintain the shape and integrity of the regenerating bone. In addition, the mesh may promote better integration of the graft material with the host bone, enhancing the biological processes involved in bone repair, such as angiogenesis [24, 25] and osteoconduction [26].

Overall, these findings suggest that the combination of a mesh with a graft is a promising approach to improving bone healing in clinical settings where enhanced bone regeneration is required. This strategy could be particularly beneficial in treating complex fractures or conditions where bone healing is compromised. Future studies should explore the long-term outcomes of this approach, as well as its applicability to different types of bone injuries and various animal models.

The results of histological studies provide valuable insights into the comparative efficacy of graft and graft + mesh materials in promoting bone regeneration and cellular proliferation. Both the graft and graft + mesh groups demonstrated significant improvements in key markers of osteogenesis, such as increased new bone formation and reduced connective tissue presence, compared with the control group. These findings highlight the effectiveness of both treatment methods in fostering an environment conducive to bone healing and regeneration. Importantly, the data show that the graft + mesh group exhibited superior outcomes in terms of new bone formation compared with the graft group. This suggests that the mesh material provided a more favorable structural scaffold for osteogenic processes than the graft material alone.

The enhanced bone formation in the graft + mesh group could be attributed to its ability to offer greater mechanical stability and better integration with the surrounding tissue [16], facilitating more efficient osteoconduction [27] and osteoinduction [28]. In addition, the mesh's open structure might have promoted better nutrient and cellular exchange, further supporting bone regeneration [29].

The increased osteoblast and osteocyte counts in both the graft and graft + mesh groups compared with the control group reinforce the conclusion that both materials support active bone formation. However, the graft + mesh group again showed significantly higher counts of these bone-forming cells than the graft group, indicating a more robust bone remodeling process. This higher cellular activity suggests that the mesh not only supports bone formation but also accelerates the overall bone healing process by stimulating cellular proliferation and differentiation [30].

Interestingly, the study found no significant differences in capillary, adipose, and dead bone areas between the groups, indicating that while the graft and mesh materials significantly influenced osteogenic parameters, they did not notably affect vascularization, fat tissue presence, or dead bone areas. This might suggest that the primary mechanism of action for both the graft and mesh materials is through direct interaction with bone-forming cells rather than through angiogenic or adipose-modulating pathways [31].

The lack of significant differences in osteoclast counts between the groups further supports the idea that the primary effect of the treatments was to enhance bone formation rather than bone resorption. This balance between bone formation and resorption is crucial for successful bone regeneration [32], and the absence of elevated osteoclast activity suggests that neither the graft nor the mesh material induced excessive bone breakdown [33], ensuring that the newly formed bone is stable and functional [34].

### 4.1. The Limitations of the Study

This study, while informative, has several limitations that should be acknowledged. The relatively small sample size may limit the statistical power and broader applicability of the findings. Although the rat radius defect model is well established for studying bone regeneration, there are inherent differences between animal models and human physiology that may affect translational relevance. In addition, the study focused on early-stage healing and did not assess long-term outcomes, which are important for evaluating the durability and stability of the regenerated bone. Another limitation is the absence of molecular analyses; while radiographic, CT, and histological assessments provided substantial information, incorporating molecular markers of bone remodeling would have offered more comprehensive insights into the healing process. Furthermore, all evaluations were conducted at a single time point, restricting the ability to observe the progression of bone regeneration over time. Future studies could address this by incorporating longitudinal analyses at multiple time points. The addition of tartrate-resistant acid phosphatase (TRAP) staining to evaluate osteoclast activity would also provide valuable information on bone remodeling dynamics. Likewise, obtaining radiographs or CT scans on day 0 or day 1 postsurgery would offer essential baseline data to strengthen the interpretations and enhance the study's rigor.

Addressing these limitations in future research will be critical for further validating and expanding upon the findings presented in this study.

## 5. Conclusion

In conclusion, this study highlights the superiority of the mesh scaffold over the graft material in promoting bone regeneration, as evidenced by enhanced new bone formation and higher osteoblast and osteocyte counts. These findings underscore the potential of mesh materials as a more effective solution for bone tissue engineering and regenerative medicine. Future research could focus on optimizing mesh design and composition to further enhance its osteogenic potential and explore its long-term effects on bone healing. In addition, investigating the interplay between osteogenesis and angiogenesis in the context of these materials could provide further insights into improving clinical outcomes for patients with bone injuries or defects.

## Figures and Tables

**Table 1:** Computed tomography (CT) scan based histomorphometry of fractured femur: 6 weeks postfracture.

	Control	Graft	Graft + mesh
TV (mm^3^)	250.3 ± 4.2	274.6 ± 3.6	285.8 ± 3.9^∗∗^
BV (mm^3^)	10.3 ± 2.9	18.4 ± 2.1	24.6 ± 2.5^∗^
BV/TV	0.041 ± 0.005	0.067 ± 0.005	0.086 ± 0.005^∗^
Conn	8.2 ± 1.9	12.1 ± 2.2	16.7 ± 1.8^∗^
Tb.N	1.1 ± 0.3	1.8 ± 0.2	2.5 ± 0.2^∗^
Tb.Th (μm)	45.1 ± 2.9	60.8 ± 3.5	72.1 ± 2.9^∗∗^
Tb.Sp (mm)	0.38 ± 0.11	0.51 ± 0.14	0.83 ± 0.15^∗^

*Note:* Data are presented as the mean ± standard deviation. BV: new bone volume, Conn: Total connectivity, TV: total volume of callus.

Abbreviations: Tb.N, trabecular number; Tb.Sp, trabecular spacing; Tb.Th, trabecular thickness.

^∗^ *p* < 0.05.

^∗∗^ *p* < 0.01, versus graft.

## Data Availability

The data that support the findings of this study are available from the corresponding author upon reasonable request.
